# A Serendipitous Journey Leading to My Love of Dendritic Patterns and Chemistry

**DOI:** 10.3390/molecules23040824

**Published:** 2018-04-04

**Authors:** Donald A. Tomalia

**Affiliations:** 1 Department of Chemistry, University of Pennsylvania, Philadelphia, PA 19104, USA; 2 Department of Physics, Virginia Commonwealth University, Richmond, VA 23284, USA; 3 National Dendrimer & Nanotechnology Center, NanoSynthons LLC, 1200 N. Fancher Avenue, Mt. Pleasant, MI 48858, USA; donald.tomalia@gmail.com; Tel.: +1-989-317-3737

As the oldest of four Midwestern boys who were offsprings of an accountant and a housewife, each with less than a formal high school degree, we were blessed to have such parents. They provided invaluable nurturing, guidance, and wisdom. Their parental nurturing was no surprise; it was a standard legacy taught by my grandparents as immigrants from Czechoslovakia in the early 1900s. However, their extraordinary wisdom and guidance could not be so easily rationalized, especially based on their meager academic pedigrees.

More specifically, my father regularly shared with me his deep interest as well as the importance of understanding natural patterns (i.e., periodicities); whether it was dealing with the weather, best fishing dates, or seashell morphologies. That unarguably influenced my keen interest in trees, dendritic patterns, and architectures which indeed emerged later in my chemistry adventure. He frequently told me that a clear understanding of fundamental patterns always rewarded the beholder with a leveraged comprehension of some hidden order within any area of chaos. That lesson became abundantly clear to me later as I pondered the power of Mendeleev’s Periodic Tables, the possibility of similar periodic order in well-defined nanoscale structures, the purpose of tree branching, and the pervasive presence of dendritic patterns throughout nature.

In the case of my mother, it was the more personal understanding of her oldest son’s personality that truly remained with me, even eight decades later. This is readily summed up in the following advice she shared with me at a very early age: “Son, your robust curiosity leads you into many fascinating areas; however, my biggest worry is that you will become hopelessly bored and disappointed when you feel you have all the answers.” In paraphrased form, she frequently reminded me that my true lifetime challenge for happiness and fulfillment would be: “To pick a career that presented an endless list of questions/mysteries that could never be totally answered and with goals that I may never completely attain”.

In my youth, I never quite appreciated her wisdom. However, my nearly six decades in chemistry have truly delivered on that challenge. I have yet to exhaust my long list of chemical mysteries and questions to be answered, which has left me too busy to be bored. For many individuals, such a career would actually be a curse; however, for me, it has turned out to be an amazing blessing.

Needless to say, I must add to this blessing my very supportive family, a long list of unselfish mentors (i.e., Prof. H. Blecker, University of Michigan; Prof. H. Heine, Bucknell University; Prof. H. Hart, Michigan State University; Prof. N.J. Turro, Columbia University; Prof. W.A. Goddard, Cal. Tech., plus countless others), as well as many loyal friends and invaluable colleagues, all of whom have enriched my life.

Now you may begin to understand, even after eight decades, how this serendipitous journey has truly led to my relentless love of chemistry, and is still going strong.

